# Glycated Hemoglobin A_1c_ Time in Range and Dementia in Older Adults With Diabetes

**DOI:** 10.1001/jamanetworkopen.2024.25354

**Published:** 2024-08-02

**Authors:** Patricia C. Underwood, Libin Zhang, David C. Mohr, Julia C. Prentice, Richard E. Nelson, Andrew E. Budson, Paul R. Conlin

**Affiliations:** 1 William F. Connell School of Nursing, Boston College, Boston, Massachusetts; 2 Veterans Affairs Boston Healthcare System, Boston, Massachusetts; 3 Center for Healthcare Organization & Implementation Research, Veterans Affairs, Boston, Massachusetts; 4 National Center for Organizational Development, Veterans Health Administration, Cincinnati, Ohio; 5 Department of Health Law, Policy, and Management, School of Public Health, Boston University, Boston, Massachusetts; 6 Boston University School of Medicine, Boston, Massachusetts; 7 Department of Internal Medicine, University of Utah Health, Salt Lake City; 8 Veterans Affairs Salt Lake City Healthcare System, Salt Lake City, Utah; 9 Harvard Medical School, Boston, Massachusetts

## Abstract

**Importance:**

Individuals with diabetes commonly experience Alzheimer disease and related dementias (ADRD). Factors such as hypoglycemia, hyperglycemia, and glycemic variability have been associated with increased risk of ADRD. Traditional glycemic measures, such as mean glycated hemoglobin A_1c_ (HbA_1c_), may not identify the dynamic and complex pathophysiologic factors in the association between diabetes and ADRD. The HbA_1c_ time in range (TIR) is a previously developed measure of glycemic control that expresses HbA_1c_ stability over time within specific ranges. This measure may inform the current understanding of the association between glucose levels over time and ADRD incidence.

**Objective:**

To examine the association between HbA_1c_ TIR and incidence of ADRD in older veterans with diabetes.

**Design, Setting, and Participants:**

The study sample for this cohort study was obtained from administrative and health care utilization data from the Veterans Health Administration and Medicare from January 1, 2004, to December 31, 2018. Veterans 65 years or older with diabetes were assessed. Participants were required to have at least 4 HbA_1c_ tests during the 3-year baseline period, which could start between January 1, 2005, and December 31, 2014. Data analysis was conducted between July and December 2023.

**Main Outcomes and Measures:**

Hemoglobin A_1c_ TIR was calculated as the percentage of days during baseline in which HbA_1c_ was in individualized target ranges based on clinical characteristics and life expectancy, with higher HbA_1c_ TIR viewed as more favorable. The association between HbA_1c_ TIR and ADRD incidence was estimated. Additional models considered ADRD incidence in participants who were above or below HbA_1c_ target ranges most of the time.

**Results:**

The study included 374 021 veterans with diabetes (mean [SD] age, 73.2 [5.8] years; 369 059 [99%] male). During follow-up of up to 10 years, 41 424 (11%) developed ADRD. Adjusted Cox proportional hazards regression models showed that lower HbA_1c_ TIR was associated with increased risk of incident ADRD (HbA_1c_ TIR of 0 to <20% compared with ≥80%: hazard ratio, 1.19; 95% CI, 1.16-1.23). Furthermore, the direction of out-of-range HbA_1c_ levels was associated with incident ADRD. Having greater time below range (≥60%, compared with ≥60% TIR) was associated with significantly increased risk (hazard ratio, 1.23; 95% CI, 1.19-1.27). Findings remained significant after excluding individuals with baseline use of medications associated with hypoglycemia risk (ie, insulin and sulfonylureas) or with hypoglycemia events.

**Conclusions and Relevance:**

In this study of older adults with diabetes, increased HbA_1c_ stability within patient-specific target ranges was associated with a lower risk of ADRD. Lower HbA_1c_ TIR may identify patients at increased risk of ADRD.

## Key Points

**Question:**

In older individuals with diabetes, is maintaining stability of glycated hemoglobin A_1c_ levels (HbA_1c_) in individualized target ranges over time associated with lower risk of Alzheimer disease and related dementias (ADRD)?

**Findings:**

In this cohort study of 374 021 older veterans with diabetes, greater HbA_1c_ stability, as measured by HbA_1c_ time in range, was associated with a lower risk of ADRD. Furthermore, when HbA_1c_ levels were out of range, greater time below individualized HbA_1c_ target ranges was associated with increased risk of ADRD, even among individuals not prescribed agents associated with hypoglycemia.

**Meaning:**

These findings suggest that for older adults with diabetes, maintaining HbA_1c_ stability in individualized target ranges over time is associated with a lower risk of ADRD.

## Teaser

This cohort study of older individuals with diabetes examines whether maintaining stability of glycated hemoglobin A_1c_ is associated with lower risk of Alzheimer disease and related dementias.

## Introduction

Dementia is a common complication of diabetes in older adults. Epidemiologic studies indicate that the incidence of Alzheimer disease and related dementias (ADRD) is higher in individuals with diabetes compared with those without diabetes.^1,2^ The mechanisms underlying this association are complex, with studies showing associations with persistently elevated glycated hemoglobin A_1c_ (HbA_1c_),^3,4^ hypoglycemia,^5^ and glucose variability.^3,6,7^ These studies show an association between abnormal glucose levels over time and ADRD but do not define a glycemic management strategy that clinicians can use to reduce the risk of ADRD among older adults with diabetes.

Diabetes clinical guidelines recommend individualizing glycemic targets in older adults based on life expectancy, current comorbidities, and microvascular complications.^8,9,10,11^ Clinicians often strive to balance risks and benefits in older adults with diabetes by avoiding more stringent HbA_1c_ targets to reduce hypoglycemia risks, while also applying an HbA_1c_ upper limit to reduce risks of acute hyperglycemia as well as chronic diabetes complications (eg, microvascular complications and mortality).^12,13,14^

Attempts to clarify the role of glycemic excursions over time on the development of ADRD have resulted in conflicting study findings. Some studies demonstrate a U-shaped association between measures of glycemic control (eg, plasma glucose^15^ and cumulative mean HbA_1c_^3^) and dementia incidence, suggesting that glucose values at extreme low and high levels contribute to cognitive dysfunction and dementia in individuals with diabetes. Other studies suggest hypoglycemia alone results in an increased risk of dementia.^5,16,17,18^ Furthermore, a meta-analysis showed that long-term glucose variability is associated with increased risk of dementia.^19^ Given these differing results, it is possible that traditional methods of glycemic control measurement fail to capture the dynamic and time-dependent nature of the association between glycemic control and the development of ADRD in older individuals with diabetes.

We developed a measure of glycemic control over time, HbA_1c_ time in range (TIR), which has been associated with risks of several diabetes complications and mortality.^12,20,21,22^ HbA_1c_ TIR is the percentage of time during a 3-year period that HbA_1c_ levels are within individualized clinical guideline–directed targets. Higher HbA_1c_ TIR is desirable, and HbA_1c_ TIR may be a better measure of the dynamic association between glucose levels over time and ADRD incidence. This study evaluated the association between HbA_1c_ TIR and ADRD in a large nationwide population of older veterans with diabetes.

## Methods

### Study Population

The study sample was obtained from administrative and health care utilization data from the Veterans Health Administration and Medicare from January 1, 2004, to December 31, 2018. Individuals were 65 years or older and dually eligible for Veterans Health Administration and traditional Medicare benefits. A diabetes diagnosis was confirmed using at least 2 outpatient or 1 inpatient diagnosis codes (*International Classification of Diseases, Ninth Revision* [*ICD-9*] or *International Statistical Classification of Diseases and Related Health Problems, Tenth Revision* [*ICD-10*]) or documentation of a prescription for diabetes medications (Figure).^23^ Each patient had a 1-year period to assess preexisting comorbidities and complications and set the initial HbA_1c_ TIR, a 3-year baseline period during which HbA_1c_ TIR was calculated and baseline diagnoses were collected, and an outcome period that lasted until they experienced an ADRD diagnosis, died, or reached the end of 2018 (study end) (eFigure in Supplement 1). The study was reviewed and approved by the institutional review board at the Department of Veterans Affairs (VA) Boston Healthcare System and was considered exempt research; therefore, informed consent was not required. The study followed the Strengthening the Reporting of Observational Studies in Epidemiology (STROBE) reporting guideline for cohort studies. Data analysis was conducted between July and December 2023.

**Figure.**
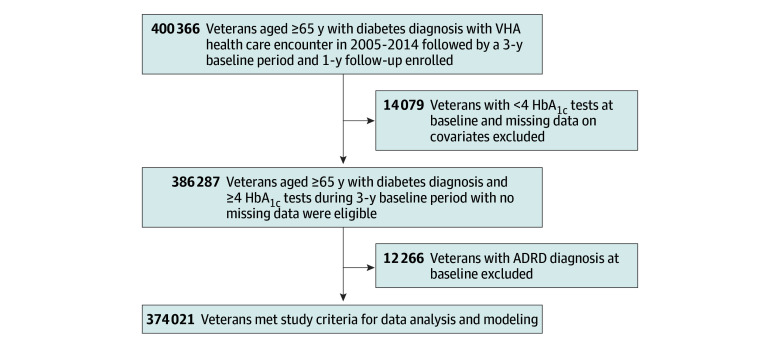
Study Flowchart ADRD indicates Alzheimer disease and related dementias; HbA_1c_, glycated hemoglobin A_1c_; VHA, Veterans Health Administration.

Participants were required to have at least 4 HbA_1c_ tests during the 3-year baseline period, which could start between January 1, 2005, and December 31, 2014. The HbA_1c_ TIR was calculated during each year of baseline using HbA_1c_ target ranges proposed in the VA/Department of Defense Clinical Practice Guideline.^10^ The 4 HbA_1c_ target ranges are 6% to 7%, 7% to 8%, 7.5% to 8.5%, and 8% to 9%. Target ranges are based on a patient’s life expectancy and the presence of comorbidities and diabetes complications.^10^ To calculate life expectancy, we used a prediction model that categorized patients into groups with mortality risks of less than 5, 5 to 9, or 10 or more years using clinical and administrative data.^24^ Diabetes complications were identified using the Diabetes Complications Severity Index^25,26^ and were updated annually in computing life expectancy. Individuals diagnosed with ADRD before the outcome period were removed from the study sample.

### Main Independent Variable

Using linear interpolation between HbA_1c_ levels and test dates, the HbA_1c_ TIR was calculated as the percentage of days during baseline that a patient’s HbA_1c_ levels were in their unique target range(s). The HbA_1c_ TIR was grouped into 20% increments. We also calculated time out of range, which was categorized into time below range (TBR) and time above range (TAR). For each patient, the sum of HbA_1c_ TIR, TBR, and TAR equaled 100%. To determine whether ADRD risks were influenced by the duration and direction of out-of-range levels, we grouped patients into 4 discrete categories: (1) HbA_1c_ TIR of 60% or greater, (2) TBR of 60% or greater, (3) TAR of 60% or greater, and (4) a mixed group (in which HbA_1c_ TIR, TBR, and TAR were all <60%) (eTable 1 in Supplement 1).

The ADRD outcomes were defined using the following *ICD-9* and *ICD-10* codes: 331.0 and G30.x for Alzheimer disease, 290.0x, 290.1x, 290.2x, 290.3x, 291.2x, 292.8x, 294.1x, 294.2x, 294.8, and F02.8x for nonspecific dementia, and 290.4x and F01.5 for vascular dementia.^27,28^

### Covariates

The analyses accounted for several baseline covariates, including patient characteristics (eg, age, self-reported race and ethnicity, self-reported gender, diabetes phenotype [type 1 or type 2], diabetes medications, and clinician type [ie, physician, nurse practitioner, or physician assistant]). Race and ethnicity are known factors associated with ADRD incidence and were included in the multivariate analyses to control for confounding effects. Time trends and facility factors were controlled with the calendar year that patients entered the outcome period and the VA medical center where the veteran received care. Comorbidities were measured during the baseline period using the Elixhauser Comorbidity Index.^25^ Comorbidities included in the analyses were cardiovascular disease, cerebrovascular disease, history of hypoglycemia (defined as a diagnosis of hypoglycemia using *ICD-9* and *ICD-10* codes), obesity, hypertension, hyperlipidemia, and hypertriglyceridemia. All were recorded as dichotomous variables based on presence or absence during the baseline period. Diabetes medications (categorized as any use) at baseline included insulin, metformin, sulfonylureas, thiazolidinediones, and other medications with less frequent use. Laboratory measurements included the number of HbA_1c_ tests during the baseline, HbA_1c_ mean during baseline, HbA_1c_ SD, serum creatinine, and blood lipids (low-density lipoprotein cholesterol and triglycerides). Clinical measures included body mass index and blood pressure. Measures were categorized based on clinically relevant cut points.

### Statistical Analysis

Descriptive analyses were conducted and reported based on ADRD status. Unadjusted as well as adjusted Cox proportional hazards regression models were used to estimate the association of HbA_1c_ TIR and time out of range categories (ie, HbA_1c_ TIR of ≥60%, TBR of ≥60%, TAR of 60% or greater, and the mixed group) and ADRD incidence. We tested assumptions of fit for the survival analysis, including examining and removing influential outliers and detecting nonlinearity in associations between the outcome and covariates. Continuous covariates that were not normally distributed were transformed to categorical variables using clinically appropriate cut points. In both models, patients were censored at death, ADRD diagnosis, or end of the study period. Fully adjusted models included all covariates. In sensitivity analyses, we evaluated associations between HbA_1c_ TIR and ADRD among individuals with and without baseline hypoglycemia and use of diabetes medications known to cause hypoglycemia (eg, sulfonylurea and insulin).^17,29^ We also conducted analyses stratified by race and ethnicity^18^ and analyzed the association between mean HbA_1c_ at the 3-year baseline and ADRD risk. We conducted a sensitivity analysis to evaluate whether the number of health care encounters (inpatient and outpatient) influenced the study findings. Because individuals are at risk for both ADRD and mortality, we also used a competing risk cause-specific Cox survival model. All statistical analyses were conducted using SAS software, version 9.4 (SAS Institute Inc). For all tests of significance, a 2-sided *P* < .05 was considered statistically significant, and we did not account for multiple comparisons. Thus, secondary outcomes and analyses should be considered hypothesis generating only.

## Results

### Demographic Information

A total of 374 021 individuals met the inclusion criteria (Table 1). The mean (SD) age at study entry was 73.2 (5.8) years, with 369 059 individuals (99%) identifying as male 4962 (1%) as female, 1326 (0.4%) as Asian, 39 711 (11%) as Black, 4193 (1%) as Hispanic, 323 883 (87%) as White, and 4908 (1%) as other (American Indian or Alaska Native, Native Hawaiian or other specific Pacific Islander, unknown by patient, or declined to answer). The mean baseline HbA_1c_ level was 7.0% (to convert to proportion of hemoglobin, multiply by 0.01). The percentages of patients in each of the 4 HbA_1c_ target ranges at the end of baseline were as follows: 6% to 7%, 94 081 patients (25.2%); 7% to 8%, 128 751 patients (34.4%); 7.5% to 8.5%, 124 844 patients (33.4%); and 8% to 9%, 26 345 patients (7.4%). During an outcome period of up to 10 years, the incidence of ADRD was 11% (n = 41 424). Demographic and clinical parameters of those who did or did not develop ADRD are given in Table 1.

**Table 1.** Baseline Characteristics of Study Participants^a^

Characteristic	Dementia (n = 41 424)	No dementia (n = 332 597)	Total group (N = 374 021)
Age, mean (SD), y	73.1 (5.8)	74.3 (5.8)	73.2 (5.8)
Sex			
Female	650 (1)	4312 (1)	4962 (1)
Male	40 774 (99)	328 285 (99)	369 059 (99)
Race			
Asian	188 (0.5)	1138 (0.3)	1326 (0.4)
Black	6145 (15)	33 566 (10)	39 711 (11)
Hispanic	762 (2)	3431 (1)	4193 (1)
White	33 827 (82)	290 056 (87)	323 883 (87)
Other^b^	502 (1)	4406 (1)	4908 (1)
CAD at baseline			
Yes	27 610 (67)	227 722 (69)	255 332 (68)
No	13 814 (33)	104 875 (31)	118 689 (32)
CVD at baseline			
Yes	12 697 (31)	93 950 (28)	106 647 (28)
No	28 727 (69)	238 647 (72)	267 374 (72)
Hypoglycemia at baseline			
Yes	2863 (7)	22 210 (7)	25 073 (7)
No	38 561 (93)	310 387 (93)	348 948 (93)
DCSI			
0	6448 (16)	55 374 (16)	61 822 (17)
1-2	14 710 (36)	114 421 (34)	129 131 (35)
3-5	15 559 (38)	119 650 (36)	135 209 (36)
6-8	4289 (10)	38 688 (12)	42 977 (11)
≥9	418 (1)	4464 (1)	4882 (1)
Insulin use at baseline			
Yes	8936 (22)	59 152 (18)	68 088 (18)
No	32 488 (78)	273 445 (82)	305 933 (82)
Metformin use at baseline			
Yes	19 159 (46)	131 975 (40)	151 134 (40)
No	22 265 (54)	200 622 (60)	222 887 (60)
Thiazolidinediones use at baseline			
Yes	5720 (14)	36 593 (11)	42 313 (11)
No	35 704 (86)	296 004 (89)	331 708 (89)
Sulfonylurea use at baseline			
Yes	20 312 (49)	132 540 (40)	152 852 (41)
No	21 112 (51)	200 057 (60)	221 169 (59)
HbA_1c_ mean (SD), %	7.0 (1.0)	6.9 (1.0)	6.9 (0.97)
HbA_1c_ SD, mean (SD), %	0.53 (0.46)	0.51 (0.45)	0.51 (0.45)
No. of HbA_1c_ tests, mean (SD)	18.5 (8.2)	17.3 (7.1)	17.4 (7.2)
Systolic blood pressure, mean (SD), mm Hg	134 (11.3)	134 (11.7)	134 (11.6)
Diastolic blood pressure, mean (SD), mm Hg	70.2 (7.1)	70.6 (7.4)	70.6 (7.3)
BMI, mean (SD)	29.6 (4.9)	30.4 (5.2)	30.3 (5.1)
Triglycerides, mean (SD), mg/dL	151.5 (86.7)	154.6 (85.9)	154.3 (86.0)
LDL, mean (SD), mg/dL	93.4 (31.7)	92.1 (31.8)	92.2 (31.8)

Abbreviations: BMI, body mass index (calculated as weight in kilograms divided by height in meters squared); CAD, cardiovascular disease; CVD, cerebrovascular disease; DCSI, Diabetes Complications Severity Index; HbA_1c_, glycated hemoglobin A_1c_; LDL, low-density lipoprotein.

SI conversion factors: To convert HbA_1c_ to proportion of hemoglobin, multiply by 0.01; triglycerides to millimoles per liter, multiply by 0.0113; and LDL to millimoles per liter, multiply by 0.0259.

^a^ All values are presented as No. (%) unless otherwise indicated.

^b^ Other includes American Indian or Alaska Native, Native Hawaiian or other specific Pacific Islander, unknown by patient, or declined to answer.

### HbA_1c_ TIR and ADRD Incidence

In unadjusted analyses, patients with lower HbA_1c_ TIR had higher ADRD risk. When compared with an HbA_1c_ TIR of 80% or greater, patients with an HbA_1c_ TIR of 0 to less than 20% had the highest risk (hazard ratio [HR], 1.59; 95% CI, 1.54-1.64; *P* < .001) (Table 2). In Cox proportional hazards regression models that controlled for all covariates, the HbA_1c_ TIR remained significantly associated with ADRD risk (HR, 1.19; 95% CI, 1.16-1.23).

**Table 2.** HbA_1c_ TIR Increments and Dementia Incidence

HbA_1c_ TIR	Unadjusted model		Fully adjusted model^a^	
	HR (95% CI)	*P* value	HR (95% CI)	*P* value
80% to 100% (n = 63 837)	1.00 [Reference]	NA	1.00 [Reference]	NA
60% to <80% (n = 55 838)	1.19 (1.15-1.23)	<.001	1.07 (1.03-1.11)	<.001
40% to <60% (n = 48 618)	1.26 (1.22-1.31)	<.001	1.06 (1.02-1.10)	<.001
20% to <40% (n = 63 807)	1.37 (1.33-1.42)	<.001	1.14 (1.09-1.18)	<.001
0 to <20% (n = 141 921)	1.59 (1.54-1.64)	<.001	1.19 (1.16-1.23)	<.001

Abbreviations: HbA_1c_, glycated hemoglobin A_1c_; HR, hazard ratio; NA, not applicable; TIR, time in range.

^a^ Covariates included in the fully adjusted model include age, sex, hypoglycemic events, Diabetes Complication Severity Index score, marital status, race, class of diabetes medication, statin use, mean HbA_1c_ during 3-year baseline period, number of HbA_1c_ tests during the 3-year baseline period, HbA_1c_ SD during the 3-year baseline period, history of heart disease, history of cerebrovascular disease, hypertension diagnosis, blood lipids (low-density lipoprotein category and triglyceride category) during 3-year baseline period, body mass index category, kidney function (creatinine category during baseline), smoking status, year entered study, primary care clinician type (ie, physician, nurse practitioner, or physician assistant), and Veterans Affairs station (geographic location).

### HbA_1c_ Time Out of Range of 60% or Greater and ADRD Incidence

Unadjusted analyses showed that when HbA_1c_ levels were mostly out of range, an HbA_1c_ TBR of 60% or greater was associated with the highest risk of ADRD (HR, 1.48; 95% CI, 1.45-1.52) (Table 3; eTable 1 in Supplement 1). After adjusting for all covariates, an HbA_1c_ TBR of 60% or greater remained significantly associated with ADRD risk when compared with an HbA_1c_ TIR of 60% or greater (HR, 1.23; 95% CI, 1.16-1.27). No association was found with incidence of ADRD in an HbA_1c_ TAR of 60% or greater (HR, 0.96; 95% CI, 0.91-1.00) and the mixed group (HR, 1.03; 95% CI, 1.00-1.06).

**Table 3.** HbA_1c_ TIR and Time Out-of-Range Categories (≥60%) and Dementia Incidence

HbA_1c_ range category	Unadjusted model		Fully adjusted model^a^	
	HR (95% CI)	*P* value	HR (95% CI)	*P* value
TIR ≥ 60% (n = 119 675)	1.00 [Reference]	NA	1.00 [Reference]	NA
TBR ≥ 60% (n = 158 697)	1.48 (1.45-1.52)	<.001	1.23 (1.19-1.27)	<.001
TAR ≥ 60% (n = 35 022)	1.09 (1.05-1.13)	<.001	0.96 (0.91-1.00)	.06
Mixed groups (n = 60 627)	1.21 (1.18-1.25)	<.001	1.03 (1.00-1.06)	.10

Abbreviations: HbA_1c_, glycated hemoglobin A_1c_; HR, hazard ratio; NA, not applicable; TAR, time above range; TBR, time below range; TIR, time in range.

^a^ Covariates included in the fully adjusted model include age, sex, hypoglycemic events, Diabetes Complication Severity Index score, marital status, race, class of diabetes medication, statin use, mean HbA_1c_ during 3-year baseline period, number of HbA_1c_ tests during the 3-year baseline period, HbA_1c_ SD during the 3-year baseline period, history of heart disease, history of cerebrovascular disease, hypertension diagnosis, blood lipids (low-density lipoprotein category and triglyceride category) during 3-year baseline period, body mass index category, kidney function (creatinine category during baseline), smoking status, year entered study, primary care clinician type (ie, physician, nurse practitioner, or physician assistant), and Veterans Affairs station (geographic location).

### Sensitivity Analyses

We evaluated the association between HbA_1c_ TIR and HbA_1c_ TBR with incident ADRD among those at risk for or having hypoglycemia during baseline. We removed individuals from the study population who were using insulin and/or sulfonylureas or had documented hypoglycemia events during baseline. Each decrement in HbA_1c_ TIR and the TBR of 60% or greater subgroup remained significantly associated with incident ADRD (Table 4). We stratified the population by differing racial and ethnic groups (eTable 2 in Supplement 1).^18^ The HbA_1c_ TIR remained associated with incident ADRD regardless of race (HbA_1c_ TIR of 0%-20% for the Black population: HR, 1.31; 95% CI, 1.20-1.43).

**Table 4.** HbA_1c_ TIR and Out-of-Range Categories (≥60%) and Dementia Incidence Among Patients Without a History of Hypoglycemia or Use of Sulfonylureas or Insulin During Baseline

HbA_1c_ range category	Unadjusted model		Fully adjusted model^a^	
	HR (95% CI)	*P* value	HR (95% CI)	*P* value
**Model 1: HbA_1c_ TIR**				
80% to 100% (n = 36 551)	1.00 [Reference]	NA	1.00 [Reference]	NA
60% to <80% (n = 25 376)	1.18 (1.11-1.25)	<.001	1.14 (1.08-1.21)	<.001
40% to <60% (n = 17 398)	1.15 (1.08-1.23)	<.001	1.14 (1.06-1.22)	<.001
20% to <40% (n = 26 647)	1.35 (1.27-1.42)	<.001	1.21 (1.17-1.32)	<.001
0 to <20% (n = 69 926)	1.79 (1.71-1.87)	<.001	1.28 (1.22-1.35)	<.001
**Model 2: HbA_1c_ TOR**				
TIR ≥ 60% (n = 61 927)	1.00 [Reference]	NA	1.00 [Reference]	NA
TBR ≥ 60% (n = 83 925)	1.69 (1.63-1.75)	<.001	1.23 (1.18-1.29)	<.001
TAR ≥ 60% (n = 9609)	0.72 (0.66-0.78)	<.001	0.97 (0.87-1.07)	.50
Mixed group (n = 20 437)	1.09 (1.03-1.15)	<.001	1.08 (1.01-1.14)	.02

Abbreviations: HbA_1c_, glycated hemoglobin A_1c_; NA, not applicable; TAR, time above range; TBR, time below range; TIR, time in range; TOR, time out of range.

^a^ Covariates included in the fully adjusted model include age, sex, hypoglycemic events, Diabetes Complication Severity Index score, marital status, race, class of diabetes medication, statin use, mean HbA_1c_ during 3-year baseline period, number of HbA_1c_ tests during the 3-year baseline period, HbA_1c_ SD during the 3-year baseline period, history of heart disease, history of cerebrovascular disease, hypertension diagnosis, blood lipids (low-density lipoprotein category and triglyceride category) during 3-year baseline period, body mass index category, kidney function (creatinine category during baseline), smoking status, year entered study, primary care clinician type (ie, physician, nurse practitioner, or physician assistant), and Veterans Affairs station (geographic location).

We also analyzed the association between mean HbA_1c_ and ADRD risk. Participants were categorized based on mean HbA_1c_ during baseline (<6%, 6%-6.9%, 7%-7.9% [reference], 8%-8.9%, 9%-9.9%, ≥10%). In unadjusted and fully adjusted models, lower mean HbA_1c_ (<6%: HR, 1.16; 95% CI, 1.17-1.40) and higher mean HbA_1c_ (≥8.0%) were significantly associated with increased risk of ADRD (eTable 3 in Supplement 1).

Results did not change in models that included the number of health care encounters (inpatient and outpatient) (HbA_1c_ TIR of 0-20%: HR, 1.16; 95% CI, 1.12-1.20) (eTables 4 and 5 in Supplement 1). Finally, in models that predicted ADRD with the competing risk of mortality, HbA_1c_ TIR and TBR of 60% or greater remained significantly associated with increased incident ADRD (eTable 6 in Supplement 1).

## Discussion

In this large sample of older veterans with diabetes, we found that HbA_1c_ stability within individualized target ranges is associated with a lower risk of ADRD. This association exists independent of known variables associated with ADRD incidence, including hypoglycemia, age, race, and medication use, as well as history of cerebrovascular and cardiovascular disease. When HbA_1c_ was mostly out of range, an HbA_1c_ TBR of 60% or greater was associated with increased risk of ADRD. This finding remained significant after removing individuals who experienced hypoglycemia and/or used diabetes medications associated with hypoglycemia risk during the 3-year baseline period. These findings suggest that in older adults with diabetes, HbA_1c_ stability over time within patient-specific targets is associated with ADRD risk, and having more time with HbA_1c_ levels below an individual’s clinically appropriate target range is associated with increased risk.

These results support work demonstrating an association between greater HbA_1c_ TIR and reduced incidence of diabetes complications and mortality.^12,20,21,22,30^ An HbA_1c_ TIR is a measure of glycemic stability over time that incorporates upper and lower limits based on a patient’s unique characteristics and provides additional information when compared with other measures of glycemic management over time. Mean HbA_1c_ does not express variability, HbA_1c_ SD is independent of mean HbA_1c_, and neither addresses HbA_1c_ trends over time.^20^ The HbA_1c_ TIR captures all these in a single measure. The HbA_1c_ TIR is different from glucose TIR, which is obtained from continuous glucose monitoring. Glucose TIR identifies the percentage of time an individual’s glucose is in a target range (ie, 70-180 mg/dL [to convert to mmol/L, multiply by 0.0555]) during a 2- to 12-week period. Glucose TIR does not individualize ranges based on a patient’s age, comorbidities, and functional status^31^ and does not measure glucose variability for a longer period. The HbA_1c_ TIR measures glycemic management during 3 years and uses individualized HbA_1c_ targets.^9,10,20^

Our findings further define the potential role of glycemic excursions and risks of developing ADRD. Prior studies suggest a complex association between glycemic control and incidence of ADRD, with several studies demonstrating that extreme values of hypoglycemia and hyperglycemia are associated with increased ADRD incidence.^3,5,15,17,18,32^ Indeed, a meta-analysis of 1.4 million patients with diabetes demonstrated that hypoglycemic episodes were associated with a significant risk of dementia.^33^ Furthermore, higher glucose variability and chronic hyperglycemia increased the risk of ADRD incidence.^3,34^ Our results confirm that extremes of glucose levels, as measured by average HbA_1c_, are associated with an increase in dementia incidence. Furthermore, our results clarify the association between glycemic control and dementia incidence by showing a significant association between HbA_1c_ stability and reduced dementia incidence. These results suggest that in addition to preventing hypoglycemia, maintaining higher HbA_1c_ TIR over time may decrease dementia incidence.

We also found that greater HbA_1c_ TBR was associated with higher risk of ADRD, even after accounting for hypoglycemia events and medication use associated with hypoglycemia (ie, insulin and sulfonylureas). Hypoglycemia and the use of such medications are associated with an increased risk of ADRD.^35,36^ However, the fact that an HbA_1c_ TBR of 60% or greater is associated with greater risk of ADRD is clinically relevant. This finding suggests that in older adults with diabetes, the stability of ambient glucose levels over time may be as important as the level per se. As an example, a previous study showed that a significant decline in HbA_1c_ levels increased dementia risk in older individuals with type 2 diabetes.^37^ Persistently low glucose levels can lead to a resetting of glucose thresholds that trigger signs and symptoms of hypoglycemia and result in hypoglycemia unawareness.^38^ Furthermore, individuals with diabetes have pathophysiologic changes in the brain that lead to alterations in neurotransmitter concentrations and cognitive dysfunction.^39,40^ It is possible that glucose levels persistently below clinically relevant target ranges, even without hypoglycemia, may lead to changes that are associated with an increased risk of ADRD. Investigating this hypothesis is an area that warrants further investigation.

### Strengths and Limitations

Our study has several strengths. We used a large national sample of older veterans who were dually eligible for VA and Medicare benefits. These comprehensive data allowed us to clearly define patient characteristics and covariates associated with outcomes. Furthermore, the study design included a 3-year baseline followed by the outcomes period, which minimizes the risk of reverse causation. Additionally, use of HbA_1c_ levels obtained from electronic health records demonstrates that HbA_1c_ TIR measurements can be readily calculated at the point of care as a measure of glycemic stability.

This study also has several limitations. The study population was mostly older men, which limits the generalizability. In addition, this is an observational study, and there are unmeasured variables (eg, social determinants of health, food insecurity, diabetes self-care behaviors, and *APOE4* [OMIM 107741] mutation) that are not available in electronic health records that may confound the observed findings.^16^ Comorbidities, complications, and outcomes were determined by coding of encounters, which may be influenced by treatment setting.^41^ Study eligibility required at least 4 HbA_1c_ tests during the 3-year baseline period, which may select for individuals with greater contact with the health care system. Furthermore, although we were able to see significant associations between HbA_1c_ TIR and ADRD, dementia diagnoses often become clinically manifest over time. Longer follow-up periods may be necessary to fully define risks associated with HbA_1c_ instability. We were also unable to use gold standard measurements to confirm dementia diagnosis.^42^ The study was conducted during a period when newer classes of diabetes medications (eg, sodium-glucose cotransporter 2 inhibitors and glucagon-like peptide 1 receptor agonists) were not frequently prescribed. We were unable to analyze the influence of these specific medications, which may decrease risk of all-cause dementia.^43^ We were unable to reliably determine the duration of an individual’s diabetes diagnosis in the electronic health record system. Finally, our results do not show that prospectively maintaining higher HbA_1c_ TIR as a treatment strategy will reduce risks for ADRD.

## Conclusions

In this study of older adults with diabetes, maintaining HbA_1c_ levels in patient-specific target ranges over time was associated with a lower risk of ADRD. Higher HbA_1c_ TIR was associated with lower risk, while higher HbA_1c_ TBR was associated with higher risk of ADRD. These results affirm the benefits of applying personalized HbA_1c_ target ranges based on age, life expectancy, and comorbidities. Clinicians should work with patients to ensure HbA_1c_ stability to reduce ADRD risk in older adults with diabetes.

## Notes

Supplement 1. **eFigure.** Study Design **eTable 1.** Baseline Characteristics of the Study Population by A1c TIR and A1c TOR **eTable 2.** A1c TIR and Incident Dementia Stratified by Race **eTable 3.** A1c Mean Category and Incident Dementia **eTable 4.** A1c Time-In-Range (TIR) Increments and Dementia Incidence **eTable 5.** A1c Time-In-Range (TIR) and ≥60%Time Out-of-Range Categories and Dementia Incidence **eTable 6.** Competing Risk Analysis: A1c Time-In-Range (TIR) and ≥60%Time Out-of-Range (TOR) Categories with Dementia and Mortality Incidence

Supplement 2.
Data Sharing Statement
