# Supplementary material for: Glycated Hemoglobin A1c Time in Range and Dementia in Older Adults With Diabetes
Source: JAMA Netw Open. 2024 Aug 2;7(8):e2425354. doi: 10.1001/jamanetworkopen.2024.25354 (PMC11297381; doi:10.1001/jamanetworkopen.2024.25354)
Supplement: Supplement 1. — eFigure. Study Design eTable 1. Baseline Characteristics of the Study Population by A1c TIR and A1c TOR eTable 2. A1c TIR and Incident Dementia Stratified by Race eTable 3. A1c Mean Category and Incident Dementia eTable 4. A1c Time-In-Range (TIR) Increments and Dementia Incidence eTable 5. A1c Time-In-Range (TIR) and ≥60%Time Out-of-Range Categories and Dementia Incidence eTable 6. Competing Risk Analysis: A1c Time-In-Range (TIR) and ≥60%Time Out-of-Range (TOR) Categories with Dementia and Mortality Incidence [file jamanetwopen-e2425354-s001.pdf]

Supplementary Online Content

Underwood PC, Zhang L, Mohr DC, et al. Glycated hemoglobin A_{1c} time in range and dementia in older adults with diabetes. *JAMA Netw Open*. 2024;7(8):e2425354. doi:10.1001/jamanetworkopen.2024.25354

eFigure. Study Design

eTable 1. Baseline Characteristics of the Study Population by A1c TIR and A1c TOR

eTable 2. A1c TIR and Incident Dementia Stratified by Race

eTable 3. A1c Mean Category and Incident Dementia

eTable 4. A1c Time-In-Range (TIR) Increments and Dementia Incidence

eTable 5. A1c Time-In-Range (TIR) and ≥60%Time Out-of-Range Categories and Dementia Incidence

eTable 6. Competing Risk Analysis: A1c Time-In-Range (TIR) and ≥60%Time Out-of-Range (TOR) Categories with Dementia and Mortality Incidence

This supplementary material has been provided by the authors to give readers additional information about their work.

eFigure. Study Design

T, Time.

Figure 1: Study Design. T, time.

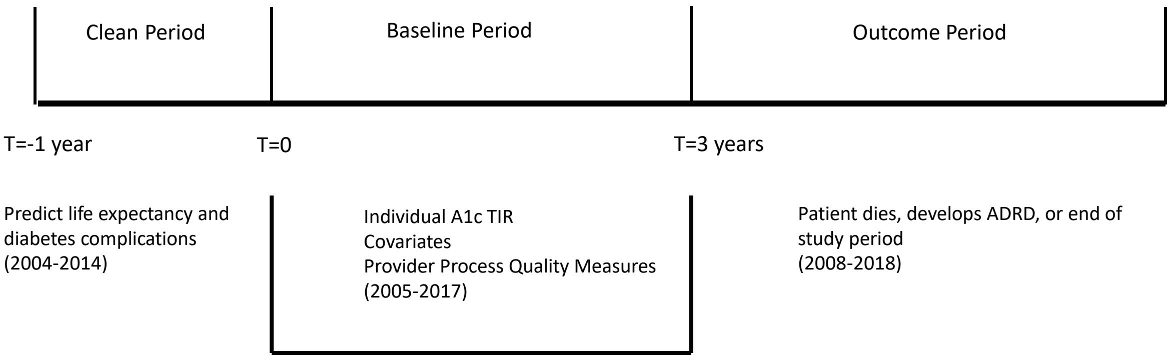

© 2024 Underwood PC et al. *JAMA Network Open.*

eTable 1. Baseline Characteristics of the Study Population by A1c TIR and A1c TOR

	**≥60% TIR**	**≥60% TBR**	**≥60% TAR**	**≥60% Mixed**
N (%)	119675 (32%)	158697 (42.4%)	35022 (9.36%)	60627 (16%)
Age at end of baseline (years)	75.4	79.5	74.3	76.3
Gender, N (%)				
Male	117966 (98.6)	156606 (98.7)	34566 (98.7)	59921 (98.8)
Female	1709 (1.4)	2091 (1.3)	456 (1.3)	706 (1.2)
Race/ethnicity, N (%)				
Asian	424 (0.4)	567 (0.4)	133 (0.4)	202 (0.3)
Black	12846 (10.7)	14508 (9.2)	5380 (15.4)	6977 (11.5)
Hispanic	1119 (0.9)	1562 (1)	663 (1.9)	849 (1.4)
Other	1798 (1.5)	1580 (1)	711 (2)	819 (1.4)
White	103488 (86.4)	140480 (88.4)	28135 (80.3)	51780 85.4)
Clinical parameters				
ADRD Incidence N (%)	12378 (10%)	18216 (11.5%)	3995 (11.4%)	6835 (11.3%)
Baseline A1c (%)	7	6.3	8.7	7.4
Number of A1c tests, median (IQR))	5 (3)	5 (2)	6 (3)	6 (2)
Body Mass Index (kg/m^2)	30.6 (5.1)	29.7 (5.2)	30.9 (5.3)	30.8 (5.4)
Diabetes Complications Severity Index (highest baseline score), n(%)				
0	32767 (27.4)	11816 (7.5)	8813 (25.2)	8426 (13.9)
1-2	47373 (40)	46180 (29.1)	14562 (41.6)	21016 (34.7)
3-5	30784 (25.7)	72692 (45.8)	8991 (25.7)	22742 (37.5)
6-8	7765(6.5)	25419 (16)	2302 (6.6)	7491 (12.4)
>=9	986 (0.8)	2590 (1.6)	354(1)	952 (1.6)

Abbreviations: SD: standard deviation, ADRD: Alzheimer’s Disease and Related Dementias, TIR: A1c Time in Range, TBR: A1c Time Below Range, TAR: A1c Time Above Range

Race/ethnicity “other” defined as American Indian or Alaska Native, Unknown by patient, Declined to Answer, Native Hawaiian or other specific Pacific Islander

© 2024 Underwood PC et al. *JAMA Network Open.*

eTable 2. A1c TIR and Incident Dementia Stratified by Race

	Black Only N=39,711		White Only N=323,883	
Model: A1c TIR	*HR* (95% *CI)*	*p*-value	*HR* (95% *CI)*	*p*-value
80-100%	1.00	-	1.00	-
60-<80%	1.16 (1.03-1.25)	0.01	1.07 (1.02-1.11)	0.002
40-<60%	1.10 (0.99-1.22)	0.07	1.05 (1.01-1.10)	0.02
20-<40%	1.28 (1.16-1.41)	<0.001	1.12 (1.07-1.16)	<0.001
0-<20%	1.31 (1.20-1.43)	<0.001	1.17 (1.13-1.22)	<0.001

© 2024 Underwood PC et al. *JAMA Network Open.*

eTable 3. A1c Mean Category and Incident Dementia

Entire Study Population		Dementia Onset		
A1c Mean Categories	N	*HR*[a]	(95% CI)	*p*-value
A1c <6%	41636	1.16	(1.08-1.24)	<0.001
A1c 6-6.9%	177749	1.03	(0.96-1.07)	0.12
A1c 7-7.9%	104654	1	-	-
A1c 8-8.9%	35298	1.1	(1.04-1.15)	0.002
A1c 9-9.9%	10265	1.29	(1.17-1.40)	<0.001
A1c >10%	4419	1.66	(1.39-1.86)	<0.001

[a]Adjusted for all covariates.

© 2024 Underwood PC et al. *JAMA Network Open.*

eTable 4. A1c Time-In-Range (TIR) Increments and Dementia Incidence

	Adjusted Model			Adjusted Model including # Health Care Encounters		
Model 1: A1c TIR	*HR*	(95% *CI)*	*p*-value	*HR*	(95% *CI)*	*p*-value
80-100% (N=63,837)	1.00	-	-	1.00	-	-
60-<80% (N=55,838)	1.07	(1.03-1.11)	0.002	1.06	(1.02-1.10)	0.002
40-<60% (N=48,618)	1.06	(1.02-1.10)	0.004	1.04	(1.01-1.09)	0.03
20-<40% (N=63,807)	1.14	(1.09-1.18)	<0.001	1.11	(1.07-1.16)	<0.001
0-<20% (N=141,921)	1.19	(1.16-1.23)	<0.001	1.16	(1.12-1.20)	<0.001

© 2024 Underwood PC et al. *JAMA Network Open.*

eTable 5. A1c Time-In-Range (TIR) and ≥60%Time Out-of-Range Categories and Dementia Incidence

	Adjusted Model			Adjusted Model including # Health Care Encounters		
	HR	95% *CI*	*p*-value	*HR*	95% *CI*	*p*-value
≥60% TIR (N=119,675)	-		-	-		-
≥60% TBR (N=158,697)	1.23	(1.19-1.27)	<0.001	1.19	(1.16-1.23)	<0.001
≥60%TAR (N=35,022)	0.96	(0.91-1.02)	0.06	0.96	(0.92-1.01)	0.07
Mixed Groups (N=60,627)	1.03	(1.00-1.06)	0.1	1.02	(0.99-1.05)	0.3

© 2024 Underwood PC et al. *JAMA Network Open.*

eTable 6. Competing Risk Analysis: A1c Time-In-Range (TIR) and ≥60%Time Out-of-Range (TOR) Categories with Dementia and Mortality Incidence

**Fully Adjusted Model**	Dementia	
**Model 1: A1c TIR**	*HR (95% CI)*	*p*-value
80-100% Reference	1	-
60-<80%	1.07 (1.03-1.11)	<0.001
40-<60%	1.06 (1.02-1.10)	0.003
20-<40%	1.14 (1.10-1.18)	<0.001
0-<20%	1.19 (1.16-1.23)	<0.001
	Dementia	
**Model 2: ≥60% TIR and TOR**	*HR (95% CI)*	*p*-value
≥60% TIR (Reference)	1	-
≥60% TBR	1.23 (1.20-1.27)	<0.001
≥60%TAR	0.94 (0.90-0.99)	0.01
Mixed Groups	1.03 (0.99-1.07)	0.07

© 2024 Underwood PC et al. *JAMA Network Open.*
