# Supplementary material for: Glycated Hemoglobin A1c Time in Range and Dementia in Older Adults With Diabetes
Source: JAMA Netw Open. 2024 Aug 2;7(8):e2425354. doi: 10.1001/jamanetworkopen.2024.25354 (PMC11297381; doi:10.1001/jamanetworkopen.2024.25354)
Supplement: Supplement 2. — Data Sharing Statement [file jamanetwopen-e2425354-s002.pdf]

Data Sharing Statement

Underwood. Glycated Hemoglobin A_{1c} Time in Range and Dementia in Older Adults With Diabetes. *JAMA Netw Open*. Published August 02, 2024.
doi:10.1001/jamanetworkopen.2024.25354

Data
Data available: No

Additional Information
Explanation for why data not available: Data is housed in the Veterans Health Administration. We do not have permission to share individual patient data.
